# *In vitro* antitumor actions of extracts from endemic plant *Helichrysum zivojinii*

**DOI:** 10.1186/1472-6882-13-36

**Published:** 2013-02-18

**Authors:** Ivana Z Matić, Ivana Aljančić, Željko Žižak, Vlatka Vajs, Milka Jadranin, Slobodan Milosavljević, Zorica D Juranić

**Affiliations:** 1 Institute of Oncology and Radiology of Serbia, Pasterova 14, 11000, Belgrade, Serbia; 2 Institute for Chemistry, Technology and Metallurgy, University of Belgrade, Njegoševa 12, 11000, Belgrade, Serbia; 3 Faculty of Chemistry, University of Belgrade, Studentski trg 16, 11000, Belgrade, Serbia

**Keywords:** *Helichrysum zivojinii*, Cytotoxicity, Cancer cells, Peripheral blood mononuclear cells, Apoptosis

## Abstract

**Background:**

The aim of this research was to determine the intensity and mechanisms of the cytotoxic actions of five extracts isolated from the endemic plant species *Helichrysum zivojinii* Černjavski & Soška (family Asteraceae) against specific cancer cell lines. In order to evaluate the sensitivity of normal immunocompetent cells implicated in the antitumor immune response, the cytotoxicity of extracts was also tested against healthy peripheral blood mononuclear cells (PBMC).

**Methods:**

The aerial parts of the plants were air-dried, powdered, and successively extracted with solvents of increasing polarity to obtain hexane, dichloromethane, ethyl-acetate, *n*-butanol and methanol extracts. The cytotoxic activities of the extracts against human cervix adenocarcinoma HeLa, human melanoma Fem-x, human myelogenous leukemia K562, human breast adenocarcinoma MDA-MB-361 cells and PBMC were evaluated by the MTT test. The mode of HeLa cell death was investigated by morphological analysis. Changes in the cell cycle of HeLa cells treated with the extracts were analyzed by flow cytometry. The apoptotic mechanisms induced by the tested extracts were determined using specific caspase inhibitors.

**Results:**

The investigated *Helichrysum zivojinii* extracts exerted selective dose-dependent cytotoxic actions against selected cancer cell lines and healthy immunocompetent PBMC stimulated to proliferate, while the cytotoxic actions exerted on unstimulated PBMC were less pronounced. The tested extracts exhibited considerably stronger cytotoxic activities towards HeLa, Fem-x and K562 cells in comparison to resting and stimulated PBMC. It is worth noting that the cytotoxicity of the extracts was weaker against unstimulated PBMC in comparison to stimulated PBMC. Furthermore, each of the five extracts induced apoptosis in HeLa cells, through the activation of both intrinsic and extrinsic signaling pathways.

**Conclusion:**

Extracts obtained from the endemic plant *Helichrysum zivojinii* may represent an important source of novel potential antitumor agents due to their pronounced and selective cytotoxic actions towards malignant cells.

## Background

Bioactive constituents of medicinal plants are in the center of attention of modern anticancer research due to their prospective roles in suppressing the different stages of malignant transformation. The antitumor potential of plant extracts and compounds could be attributed to their ability to induce changes in the regulation of target molecules in oncogenic signal transduction pathways implicated in cell growth, replication, apoptosis, as well as in angiogenesis, invasion and metastasis of cancer cells [1-4]. To evaluate the anticancer properties of novel chemotherapeutic agents, the selectivity of their actions against malignant cells in comparison to healthy non-transformed cells, especially immunocompetent cells involved in the immune control of tumor suppression, needs to be carefully examined.

*Helichrysum zivojinii* Černjavski & Soška is an endemic plant species that grows in the National Park "Galičica" in Macedonia. Some of the plant species from the large genus *Helichrysum* are used in different regions of the world in traditional medicine for treating wounds, respiratory tract infections and gastro-intestinal disorders [5-8]. This plant genus is a valuable source of several different secondary metabolites/phytochemicals, such as flavonoids, acetophenones, phloroglucinols, pyrones, diterpenes and sesquiterpenes [5]. Different morphological groups of *Helichrysum* species often display unique qualitative and quantitative chemical compositions [5]. It has been reported that extracts and individual constituents of these plants possess significant biological and pharmacological properties, including antibacterial, antiviral, antifungal, antioxidant, anti-inflammatory and antidiabetic activities [9-16]. A search through the literature suggests that plants from the genus *Helichrysum* could be a significant source of compounds with potential anticancer activities [17-20].

The main goal of this research was to investigate the cytotoxic activities of five extracts isolated as fractions from the endemic plant *Helichrysum zivojinii* towards selected human malignant cell lines. To assess the sensitivity of normal immunocompetent cells included in the antitumor immune response, the cytotoxicity of these extracts was also tested against human peripheral blood mononuclear cells (PBMC) – both unstimulated and stimulated to proliferate by the mitogen phytohemagglutinin (PHA). To elucidate the molecular mechanisms of the cytotoxic effects of the tested extracts, the distribution of target HeLa cells at specific phases of the cell cycle after the actions of these agents was also analyzed. The mode of HeLa cell death induced by the extracts was also investigated. Elucidation of the signaling pathways implicated in the induction of apoptosis by the tested extracts was conducted by identification of target caspases.

## Methods

### Plant extracts

The plant material was collected at Tomoros (ca. 1700 altitude), mountain Galičica (Macedonia) during the flowering (17 July 2010) and identified by Vlado Matevski, Institute of Biology, Faculty of Natural Sciences and Mathematics, http://Ss. Cyril and Methodius University of Skopje, where the voucher specimen is deposited at Macedonian National Herbarium (MKNH) under the number MKNH121335.

Air-dried and powdered aerial parts of *Helichrysum zivojinii* (330 g) were extracted twice with *n*-hexane in an ultrasonic bath for 45 min. The combined extracts were concentrated in a vacuum to obtain a hexane extract (4.2 g). The plant material was successively extracted in the same manner with solvents of rising polarity to obtain a dichloromethane extract (1.4 g), an ethyl-acetate extract (0.7 g), a *n*-butanol extract (5.4 g) and finally a methanol extract (12.4 g).

Stock solutions of the investigated extracts were made in dimethyl sulfoxide (DMSO) at a concentration of 5 mg/ml.

### Instrumentation and chromatographic conditions

^1^HNMR spectra were recorded with Varian Gemini 200 in CDCl_3_ and DMSO-d_6_ with TMS as an internal standard. HPLC-MS analysis was performed with an Agilent 1100 Series chromatography system equipped with a binary pump, degasser, autosampler, column Li Chrospher 100 RP 18 (250 × 4,0 mm i.d. 5 μm), and DAD detector in combination with 6210 Time of Flight MS (Agilent Technologies). The mobile phase consisted of 0.2% formic acid in water (solvent A) and 100% acetonitrile (solvent B) with the following gradient elution: 0–5 min 10–20% B, 5–10 min 20% B, 10–20 min 20–30% B, 20–30 min 30–70% B, 30–35 min 70–100% B, 35–40 min 70% B, 40–41 min 100–10% B, 41–45 min 10% B, at a flow rate of 1 ml/min. The injection volume was 10 μL, the column temperature was 25°C. The effluent was monitored with DAD (190–550 nm) and a mass detector (ESI) which operated in negative mode at atmospheric pressure; the mass range was from *m*/*z* 100–2500, with the following ESI parameters: capillary voltage: 4000 V; gas temperature: 350°C; nebulizer pressure: 45 psig; fragmentor voltage: 140 V. Mass Hunter Workstation software was used for data analysis.

### Cell culture

Human cervix adenocarcinoma HeLa, human melanoma Fem-x and human breast adenocarcinoma MDA-MB-361 cells were cultured as monolayers. Human chronic myelogenous leukemia K562 cells were grown in a suspension in nutrient medium. Cancer cell lines were obtained from the American Type Culture Collection (Manassas, VA, USA). The complete nutrient medium was RPMI 1640 supplemented with 3 mM L-glutamine, 100 μg/ml streptomycin, 100 IU/ml penicillin, 10% heat-inactivated (56°C) fetal bovine serum and 25 mM Hepes adjusted to pH 7.2 with a bicarbonate solution. The cells were grown at 37°C in an atmosphere of 5% CO_2_ and humidified air. RPMI 1640, L-glutamine and Hepes were obtained from PAA (Pasching, Austria).

### Preparation of peripheral blood mononuclear cells

Peripheral blood mononuclear cells (PBMC) were separated from whole heparinized blood of two healthy volunteers by Lymphoprep (Oslo, Norway) gradient centrifugation. Interface cells were washed three times with Haemaccel (aqueous solution supplemented with 145 mM Na^+^, 5.1 mM K^+^, 6.2 mM Ca^+^, 145 mM Cl^-^ and 35 g/l gelatin polymers, pH 7.4), counted and resuspended in nutrient medium. The protocol of the study was approved by the Ethics Committee of the Institute of Oncology and Radiology of Serbia. Written informed consent was obtained from each healthy donor.

### Treatment of cancer cell lines

HeLa (2,000 cells per well), Fem-x (2,000 cells per well), MDA-MB-361 (10,000 cells per well) were seeded into 96-well microtiter plates and 20 h later, after cell adherence, five different concentrations of the tested extracts were added to the wells. Nutrient medium was only added to the cells in the control wells. K562 cells (5,000 cells per well) were seeded 2 h before addition of the extracts. Stock solutions of plant extracts were diluted with complete nutrient medium and applied to target cells at different final concentrations that ranged from 6.25 μg/ml to 100 μg/ml for extracts 1–4, and from 12.5 μg/ml to 150 μg/ml or 200 μg/ml for extract 5. All experiments were done in triplicate. Cisplatin was used as a positive control.

### Treatment of PBMC

PBMC (150,000 cells per well) were seeded into nutrient medium or in nutrient medium enriched with (5 μg/ml) (PHA) in 96-well microtiter plates. After 2 h, five different concentrations of the plant extracts were added to the individual wells, in triplicate, except to the control wells where a nutrient medium only was added to the cells. The final concentrations of the tested extracts ranged from 12.5 μg/ml to 200 μg/ml. PHA was obtained from INEP (Belgrade, Serbia). Cisplatin was used as a positive control.

### Determination of target cell survival

Cell survival was determined by the MTT test according to the method of Mosmann [21] and modified by Ohno and Abe [22]. Briefly, after the treatment with plant extracts for 72 h, 10 μl of MTT solution (3-(4,5-dimethylthiazol-2-yl)-2,5-dyphenyl tetrazolium bromide) was added to each well. Samples were incubated for a further 4 h, followed by the addition of 100 μl of 10% SDS. Absorbance at 570 nm was measured the next day.

To quantify cell survival (S%), the absorbance of a sample with cells grown in the presence of different concentrations of the investigated agents was divided by the absorbance of the control cells grown only in the nutrient medium, and multiplied by 100. It is implied that the absorbance of the blank was always subtracted from the absorbance of the corresponding sample with target cells. The IC_50_ was defined as the concentration of the agent that inhibited cell survival by 50%, compared to the vehicle-treated control.

### Morphological evaluation of HeLa cell death

To evaluate whether the extracts from the endemic plant *Helichrysum zivojinii* induce apoptosis in HeLa cells, morphological analysis by microscopic examination of acridine orange/ethidium bromide-stained target cells was performed. HeLa cells were seeded overnight on coverslips (100,000 cells) in 2 ml of complete medium. The next day, cells were treated with plant extracts for 24 h at concentrations corresponding to IC_90_ values that were obtained after treatments that lasted 72 h. After this period, the target cells were stained with 18 μl of a mixture of the DNA dyes acridine orange and ethidium bromide (3 μg/ml AO and 10 μg/ml EB in PBS), and visualized under a fluorescence microscope using a fluorescein isothiocyanate (FITC) filter set.

### Cell cycle analysis

HeLa cells were incubated in the presence of two different concentrations (corresponding to the IC_50_ and IC_90_ values determined after 72 h) of the examined *Helichrysum zivojinii* extracts for 24, 48 and 72 h. After these incubation times, the target cells were collected, washed and fixed in 70% ethanol on ice. Samples were stored at −20°C for one week before staining. HeLa cells were washed in PBS, resuspended in 500 μl of staining solution (PBS containing RNAse A at a final concentration of 200 μg/ml, and propidium iodide (PI) at a final concentration of 20 μg/ml), and incubated for 30 min at 37°C.

Cell cycle phase distribution was determined using a FACSCalibur Flow Cytometer (BD Biosciences Franklin Lakes, NJ, USA). The data (10,000 events collected for each sample) were analyzed using CELLQuest software (BD Biosciences).

### Determination of target caspases

To identify the caspases involved in the apoptotic cell death pathway induced by the investigated extracts, the percentages of HeLa cells pretreated with caspase inhibitors in the subG1 phase were determined. HeLa cells were preincubated for 2 h with specific caspase inhibitors (at a final concentration of 40 μM). These were: Z-DEVD-FMK, a caspase-3 inhibitor, Z-IETD-FMK, a caspase-8 inhibitor and Z-LEHD-FMK, a caspase-9 inhibitor. Caspase inhibitors were purchased from R&D Systems (Minneapolis, USA). Five tested extracts were applied to the HeLa cells at concentrations that corresponded to the IC_90_ values obtained after 72 h. For each extract, one sample of HeLa cells was not treated with an inhibitor and served as a reference sample. After 24 h of incubation, cells were harvested and fixed in 70% ethanol on ice. Samples were stored at −20°C for one week before PI staining. Changes in the percentages of cells in the subG1 phase were determined using a FACSCalibur Flow Cytometer and analyzed using CELLQuest software.

## Results

### Chemical analysis of plant extracts

The hexane (1) and dichloromethane extracts (2) presented similar ^1^H NMR spectra (recorded in CDCl_3_). The signals in the ^1^H NMR spectra of both extracts pointed to a complex mixture with prevailing relatively non-polar substances (region δ 0.8–2.2). LC/DAD analysis revealed the presence of a flavonoid with the molecular formula C_18_H_16_O_7_ (344) in the hexane extract, while the flavanone naringenin and flavone apigenin were confirmed in the dichloromethane extract, also by LC/DAD analysis. Three compounds found in both extracts are presented in Table 1, with their presumed molecular formulae and measured m/z values corresponding to identified ions obtained by ESI ToF mass spectrometry. According to LC/DAD analysis, these compounds unfortunately did not absorb in the UV spectrum, suggesting the presence of structures without a chromophore. The ethyl-acetate extract (3) and *n*-butanol extracts (4) presented ^1^H NMR spectra (recorded in DMSO-d_6_) with approximately identical groups of signals in the regions δ 12.5–13.9 (hydroxy protons), δ 5.9-8.1 (protons on the aromatic ring that is substituted with one or more hydroxy groups), and δ 4.7–5.5 (group of protons corresponding to a sugar unit). The ^1^H NMR spectrum of the most abundant methanol extract (5), also recorded in DMSO-d_6_, resembles the spectra of the ethyl-acetate and *n*-butanol extracts by the presence of signals in the region δ 5.9–8.1 (aromatic protons), as well as by the presence of a signal at δ 13.9 (hydroxy proton); it differs from the last two spectra by possessing a more pronounced region at δ 4.7–5.5, which is responsible for the protons of sugar units. LC/DAD analysis and ESI ToF mass spectrometry of these three more polar extracts (3, 4 and 5) revealed quite similar constituents in each of them. Apart from chlorogenic acid, two groups of flavonoid compounds were detected: flavonoid *O-*glycosides, and flavonoid aglycons. Among the *O-*glycosides, the glucoside (or possibly galactoside) of quercetin, the glucoside of apigenin, the glucoside of kaempferol (or possibly luteolin), and another glucoside of kaempferol (possibly luteolin or 6-hydroxyapigenin), were present. Flavonoid aglycones, such as apigenin and naringenin, were detected. Phthalic acid was found in extracts 2–5.

**Table 1:** **Components of five *Helichrysum zivojinii *extracts**

**Compounds**		**Extracts**				
		**Hexane (1)**	**CH**_**2**_**Cl**_**2 **_**(2)**	**EtOAc (3)**	**BuOH (4)**	**MeOH (5)**
**1.**	C_8_H_6_O_4_ (166) Phtalic acid	–	+	+	+	+
**2.**	C_21_H_20_O_12_ (464) *O*–glc or *O*–gal of quercetin	–	–	+	+	+
**3.**	C_25_H_24_O_12_(516) chlorogenic acids	–	–	+	+	++^a^
**4.**	C_21_H_20_O_11_(448) *O*–glc of apigenin	–	–	+	++^a^	+
**5.**	C_21_H_20_O_10_(432) *O*–glc of kaempferol or luteolin	–	–	+	**+**	**+**
**6.**	C_21_H_20_O_11_(448) *O*–glc of apigenin	–	–	+	+	+
**7.**	C_19_H_30_O_14_ or C_26_H_26_O_9_ (482)	+	+	–	–	–
**8.**	C_15_H_10_O_6_(286) *O*–glc of flavonols kaempferol, luteolin or 6-hydroxyapigenin	–	–	+	+	+
**9.**	C_21_H_24_O_9_ or C_14_H_28_O_14_ (420)	+	+	–	–	–
**10.**	C_15_H_12_O_5_ (272) flavanone naringenin	–	+	+	+	+
**11.**	C_22_H_26_O_9_ or C_15_H_30_O_14_ (434)	+	+	–	–	–
**12.**	C_15_H_10_O_5_ (270) apigenin	–	+	++^a^	+	+
**13.**	C_21_H_18_O_4_ (334)	+	–	–	–	–
**14.**	C_18_H_16_O_7_ (344)	+	–	–	–	–

^a^More abundant on the expense of other fraction constituents.

### *In vitro* cytotoxic activity

**Figure 1:**
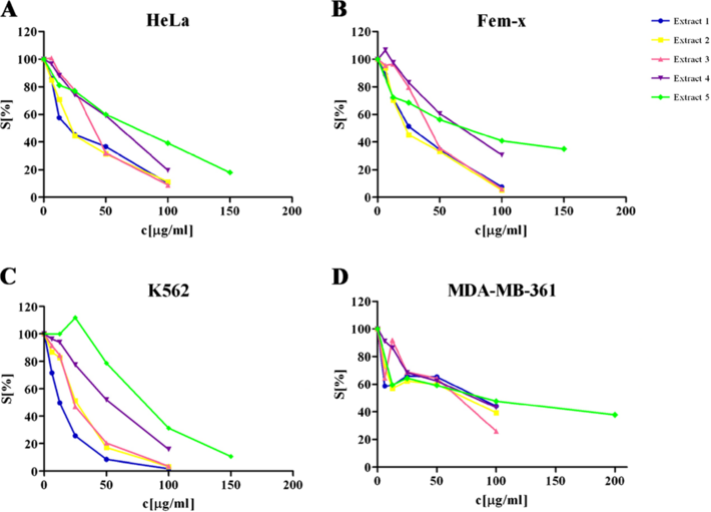
**Survival of HeLa (A), Fem-x (B), K562 (C) and MDA-MB-361 cells (D) grown for 72 h in the presence of increasing concentrations of *Helichrysum zivojinii *extracts, determined by MTT test.** Representative graphs are shown.

The cytotoxicity of the five isolated extracts was tested against selected cancer cell lines: human cervix adenocarcinoma HeLa, human melanoma Fem-x, human myelogenous leukemia K562 and human breast adenocarcinoma MDA-MB-361 cells. All investigated extracts exerted selective dose-dependent cytotoxic actions on malignant cells. The decrease in survival of target cancer cells induced by the five *Helichrysum zivojinii* extracts is shown in Figure 1 and Table 2.

**Table 2:** **Concentrations of five *Helichrysum zivojinii *extracts, which induced 50% decrease in the target cancer cell survival, determined by MTT test**

	**HeLa**	**Fem-x**	**K562**	**MDA-MB-361**
**Extract 1 IC**_**50 **_**[μg/ml]**	24.63 ± 4.12	28.85 ± 5.49	11.78 ± 0.94	81.74 ± 6.27
**Extract 2 IC**_**50 **_**[μg/ml]**	20.11 ± 4.49	23.64 ± 1.41	23.82 ± 6.54	81.74 ± 13.31
**Extract 3 IC**_**50 **_**[μg/ml]**	37.98 ± 2.33	47.04 ± 4.79	27.52 ± 4.96	79.93 ± 13.49
**Extract 4 IC**_**50 **_**[μg/ml]**	56.70 ± 6.05	74.84 ± 7.55	50.37 ± 3.28	69.96 ± 11.70
**Extract 5 IC**_**50 **_**[μg/ml]**	84.68 ± 10.39	77.29 ± 6.55	74.88 ± 7.57	94.92 ± 6.85
**Cisplatin IC**_**50 **_**[μM]**	5.60 ± 1.41	5.02 ± 0.59	5.35 ± 0.70	28.23 ± 5.04

Time of continuous agent’s action was 72 h.

In general, extracts 1 and 2 (as well as cisplatin, which served as a positive control) exhibited the highest cytotoxic actions against target malignant cell lines; extracts 3 and 4 displayed less pronounced cytotoxicity; extract 5 had the lowest cytotoxic action.

With regard to the specific sensitivities of the different cells to the cytotoxic activities of the extracts, it is important to note that K562 cells were the most sensitive to the cytotoxic actions of extracts 1 and 3. HeLa and Fem-x cells exhibited a lower sensitivity, while the sensitivity of breast cancer MDA-MB-361 cells to the toxic actions of the tested extracts was the lowest (being several times lower than that of the other cell lines to extracts 1, 2 and 3 especially).

**Figure 2:**
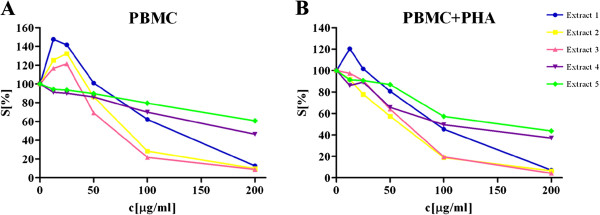
**Survival of resting PBMC (A) and PHA-stimulated PBMC (B) grown for 72 h in the presence of increasing concentrations of *Helichrysum zivojinii *extracts, determined by MTT test.** Representative graphs are shown.

Considering the possible effects of applied antitumor drugs on normal healthy immunocompetent cells, components of the antitumor immune response, their viability is significant for tumor control. For that reason, the activities of the investigated *Helichrysum zivojinii* extracts were evaluated against healthy unstimulated and PHA-stimulated PBMC (Figure 2 and Table 3). It should be noted that these extracts overall exhibited weaker cytotoxic effects against unstimulated PBMC than against stimulated PBMC. Moreover, extracts 2 and 3 exerted a more pronounced cytotoxicity against unstimulated and PHA-stimulated PBMC than extracts 1, 4 and 5.

**Table 3:** **Concentrations of five *Helichrysum zivojinii *extracts, which induced 50% decrease in target PBMC survival, determined by MTT test**

	**PBMC**	**PBMC + PHA**
**Extract 1 IC**_**50 **_**[μg/ml]**	131.59 ± 9.93	99.07 ± 8.02
**Extract 2 IC**_**50 **_**[μg/ml]**	81.74 ± 0.50	67.65 ± 11.37
**Extract 3 IC**_**50 **_**[μg/ml]**	74.82 ± 6.51	61.86 ± 5.53
**Extract 4 IC**_**50 **_**[μg/ml]**	185.17	92.20 ± 9.01
**Extract 5 IC**_**50 **_**[μg/ml]**	> 200	128.12 ± 35.46
**Cisplatin IC**_**50 **_**[μM]**	> 33.34	> 33.34

Time of continuous agent’s action was 72 h.

In order to further evaluate the anticancer potential of the extracts, the selectivity in the antitumor action against specific malignant cell line in comparison to healthy PBMC was determined as well. These data are presented in Table 4 from which it can be observed that extract 1 exhibited highly selective antitumor action, especially against K562 cells. Extract 2 also displayed good selectivity in its antitumor action.

**Table 4:** **Selectivity in the antitumor action of five *Helichrysum zivojinii *extracts**

**Selectivity coefficient in the antitumor action**	**IC**_**50**_**PBMC/ IC**_**50 **_**HeLa**	**IC**_**50**_**PBMC + PHA/ IC**_**50 **_**HeLa**	**IC**_**50**_**PBMC/ IC**_**50 **_**K562**	**IC**_**50**_**PBMC + PHA/ IC**_**50 **_**K562**
**Extract 1**	5.34	4.02	11.17	8.41
**Extract 2**	4.06	3.36	3.43	2.84
**Extract 3**	1.97	1.63	2.78	2.25
**Extract 4**	3.27	1.63	3.68	1.83
**Extract 5**	> 2.36	1.51	2.67	1.71

### Morphological analysis of HeLa cell death mode

**Figure 3:**
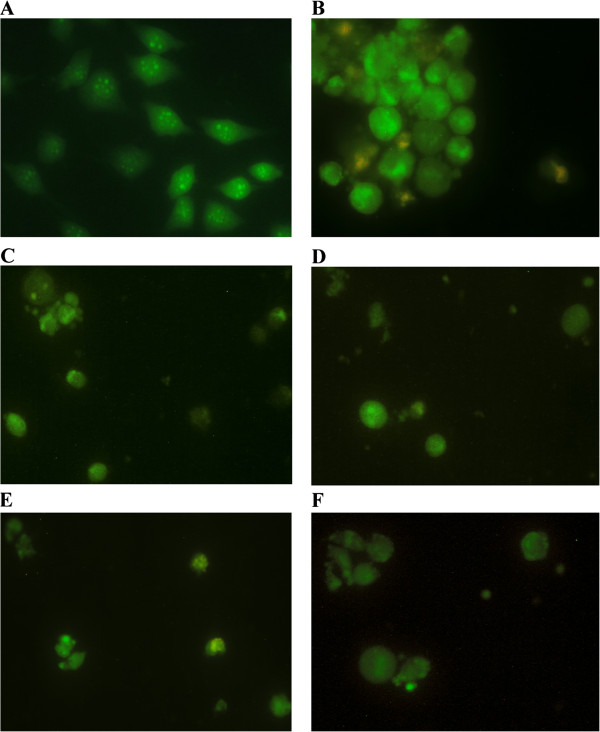
**Photomicrographs of acridine orange/ethidium bromide-stained control HeLa cells (A), and HeLa cells treated with different *Helichrysum zivojinii *extracts for 24 h (extracts 1–5, photomicrographs B-F, consecutively).**

In order to determine whether the investigated plant extracts have pro-apoptotic activities, we performed morphological analysis by fluorescent microscopy of acridine orange/ethidium bromide-stained HeLa cells, exposed to the extracts. Microscopic examination revealed that all five extracts applied at IC_90_ concentrations induced apoptosis in target HeLa cells after 24 h treatment (Figure 3). The morphological characteristics of apoptotic cell death, such as cell shrinkage, condensation and even fragmentation of nucleus, as well as the presence of orange-red stained cells at late stages of apoptosis or secondary necrosis (the latter was observed in extract 1-treated HeLa cells) and apoptotic bodies. These analyses confirmed that the cytotoxicity of the *Helichrysum zivojinii* extracts is based on their prominent pro-apoptotic effects.

### Analysis of changes in cell cycle phase distribution

**Figure 4:**
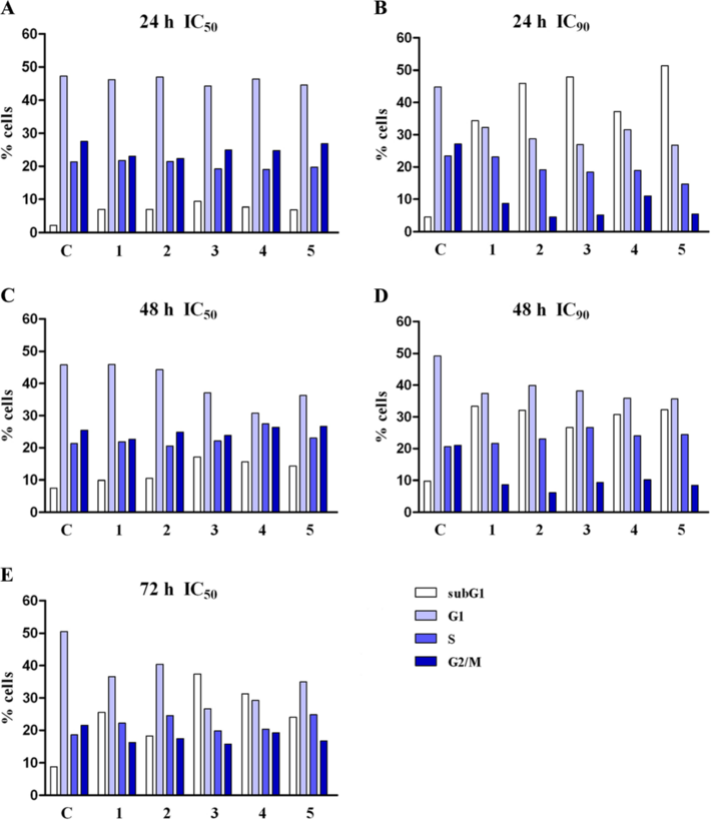
**Changes in the cell cycle phase distribution of HeLa cells induced by the *Helichrysum zivojinii *extracts after 24 (A,B), 48 (C,D) and 72 h (E) treatment (applied concentrations of tested extracts corresponded to IC**_**50 **_**and IC**_**90 **_**values determined for 72 h).** (C- control HeLa cells, 1 – 5 – corresponding extracts are numbered consecutively). Representative graphs are shown.

Examination of changes in the cell cycle phase distribution of HeLa cells treated with these extracts for 24, 48 and 72 h was done to elucidate the mechanisms of the observed cytotoxic actions (Figure 4). Results from this analysis showed a time - dependent increase in the percentages of HeLa cells in the subG1 phase after exposure to an IC_50_ concentration of all of the tested extracts. Additionally, exposure to extracts at IC_90_ concentrations induced significant increases in the percentages of cells in the subG1 phase 24 h after exposure. It should be mentioned that the investigated extracts induced a slight accumulation of HeLa cells in the S phase after 72 h. Examination of the cell cycle changes that were induced after exposure for 72 h to IC_90_ for each extract was not performed because at this time point and at this extract concentration low numbers of mostly dead or dying cells were present in the sample.

**Figure 5:**
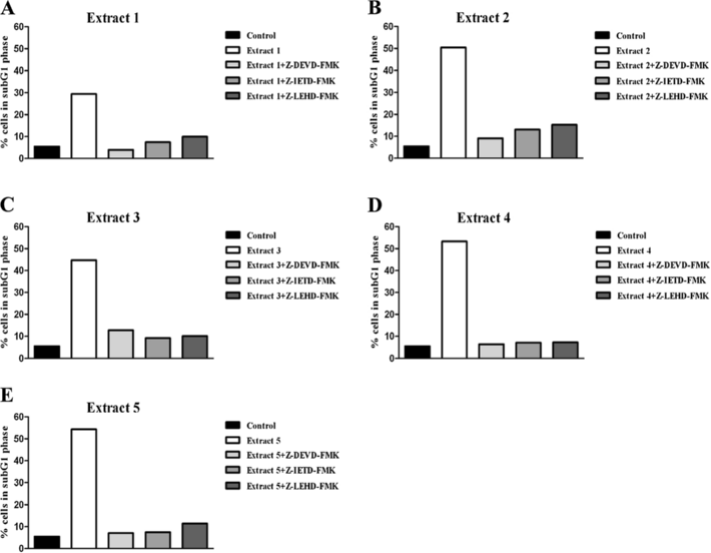
**Effects of specific caspase inhibitors on the percentages of apoptotic subG1 HeLa cells treated with *Helichrysum zivojinii *extracts for 24 h (A - extract 1, B - extract 2, C - extract 3, D - extract 4, E - extract 5).** (Z-DEVD-FMK - caspase-3 inhibitor; Z-IETD-FMK -caspase-8 inhibitor; Z-LEHD-FMK - caspase-9 inhibitor).

**Figure 6:**
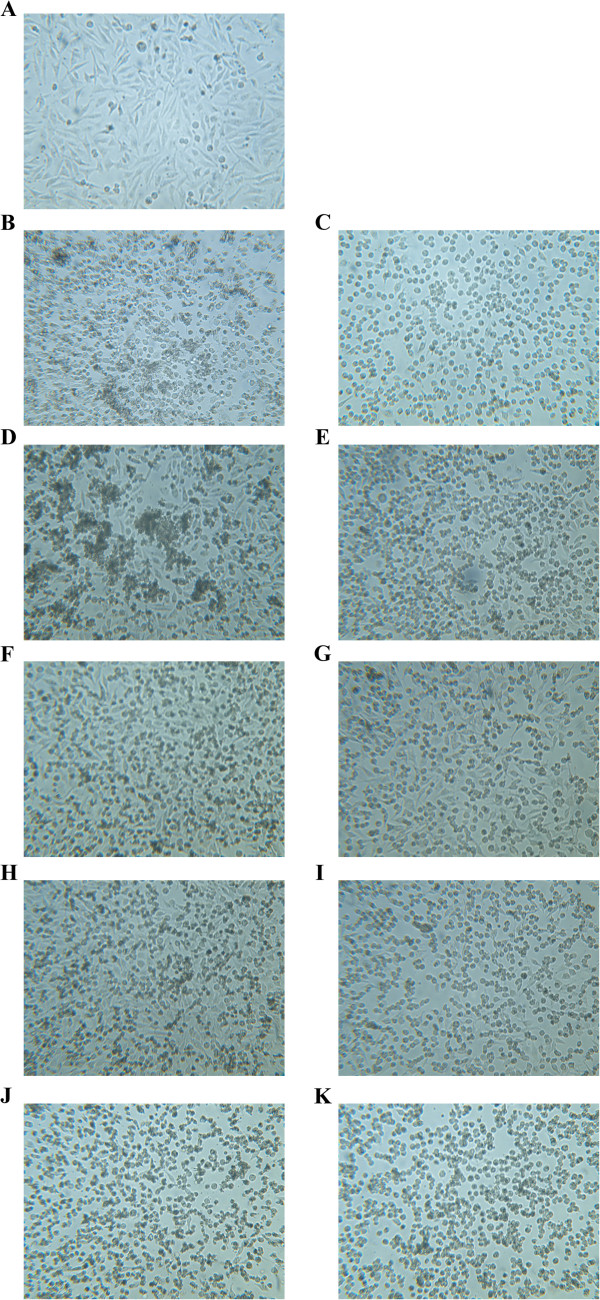
**Effects of pretreatment of HeLa cells with caspase-3 inhibitor (Z-DEVD-FMK), exposed to *Helichrysum zivojinii *extracts (applied concentrations of tested extracts corresponded to IC**_**90 **_**values determined for 72 h). A** – control; **B** – Extract 1, **C** – Extract 1 + Z-DEVD-FMK; **D** – Extract 2, **E** – Extract 2 + Z-DEVD-FMK; **F** – Extract 3, **G** – Extract 3 + Z-DEVD-FMK; **H** – Extract 4, **I** – Extract 4 + Z-DEVD-FMK; **J** – Extract 5, **K** – Extract 5 + Z-DEVD-FMK.

Since the *Helichrysum zivojinii* extracts exhibited the pro-apoptotic activities against cervix adenocarcinoma HeLa cells, the identification of target caspases involved in the apoptotic pathway was performed. The presence of the specific caspase inhibitors (caspase-3 inhibitor, caspase-8 inhibitor or caspase-9 inhibitor) significantly reduced the percentages of apoptotic subG1 HeLa cells treated with each of the five plant extracts, as shown in Figure 5. The effect of the caspase-3 inhibitor on HeLa cells treated with extracts is shown in Figure 6. It can be seen that there is an increase in rounded, but attached and live HeLa cells treated with the caspase-3 inhibitor before the addition of the extracts in relation to target HeLa cells only exposed to the tested extracts.

## Discussion

The plant kingdom provides a rich source of compounds with promising cancer chemopreventive and cancer therapeutic potential. The main drugs currently used in clinical practice in the treatment of malignant diseases originate from plants: vinca alkaloids, taxanes, camptothecins and epipodophyllotoxins [23]. Over the past years, the focus of modern anticancer drug discovery has been on a wide variety of natural compounds, especially on phenolic compounds. Phytochemicals have been reported to affect different intracellular signaling pathways implicated in the initiation, promotion and progression of cancer. The antitumor effects of plant constituents have been associated with the induction of carcinogen detoxifying enzymes, the scavenging of free radicals, anti-inflammatory activity, cell cycle arrest, the triggering of apoptosis, inhibition of tumor angiogenesis and invasiveness [1-4].

The antioxidant and anti-inflammatory activities of extracts and isolated compounds from plants belonging to the large genus *Helichrysum* have been well documented [9,11,15,18]. Arzanol, a phloroglucinyl α-pyrone, is a constituent of *Helichrysum italicum* that has been reported to inhibit the NF-κB transcription factor, cyclooxygenase and lipooxygenase, as well as the release of proinflammatory cytokines [11,18]. Regarding the link between inflammation and cancer, chemicals with anti-inflammatory properties targeting the molecules of signaling cascades implicated in inflammation and carcinogenesis may be useful as cancer chemopreventive drugs.

On the other hand, data about the potential anticancer activity of extracts and phytochemicals of plants from the genus *Helichrysum* are scarce. The antiproliferative effect of the ethanol extract from *Helichrysum maracandicum* towards SENCAR mouse skin transformed cells has been demonstrated [20]. This extract suppressed the expression of p38 MAP kinase. An examination of the arzanol properties showed that this compound, which was isolated from *Helichrysum italicum,* did not exert cytotoxic action against monkey VERO cells at concentrations up to 40 μM [24]. In contrast, another study showed that arzanol at a concentration of 50 μM significantly suppressed the survival of human lung carcinoma A549 cells [18]. It should be mentioned that methanolic extracts prepared from different *Helichrysum* species were found to inhibit DNA topoisomerase I [19]. Moreover, the cytotoxicity of *Helichrysum gymnocephalum* essential oil towards human breast adenocarcinoma MCF-7 cells has been documented [17].

The results presented herein demonstrate the selective dose-dependent cytotoxic actions of the five extracts isolated from the endemic plant species *Helichrysum zivojinii* against target cancer cell lines and against healthy immunocompetent PBMC that have been stimulated to proliferate, while their cytotoxic actions were not as pronounced against unstimulated PBMC. The observed selectivity in the antitumor effects of the extracts against specific malignant cell types could be attributed to the actions of different *Helichrysum zivojinii* constituents on target molecules of the signal transduction pathways that regulate cell proliferation and apoptosis. Furthermore, each of the investigated extracts exhibited considerably stronger cytotoxicity to HeLa, Fem-x and K562 cells when compared to PBMC, both resting and PHA-stimulated, which points to the cancer specificity of their actions. It is noteworthy that when these extracts were applied at concentrations that were highly cytotoxic to malignant cells, they demonstrated very low toxicity towards healthy immunocompetent PBMC, the key players in immune defenses against tumors. The good selectivity of their antitumor actions highlights the significant anticancer potential of *Helichrysum zivojinii* extracts. The prominent antitumor properties of these extracts need to be examined further in *in vivo* studies.

It should be stressed that all of these extracts exhibited weaker cytotoxic effects against unstimulated PBMC in comparison to stimulated PBMC. This finding indicates that the extracts possess the ability to inhibit the proliferation of PHA-stimulated PBMC. Thus, these agents may even suppress certain immune functions, particularly non-specific antigen stimulation. Additionally, the observed lower activities against resting PBMC than against mitogen-stimulated PBMC point to components in pathways regulating cell proliferation as the possible molecular targets of the *Helichrysum zivojinii* extracts. However, it is very important to note that when extracts 1, 2 and 3 were applied at lower concentrations, they stimulated the proliferation of resting PBMC. This growth stimulation effect of lower concentrations of extracts is in accordance with the well-known effect of very small doses of X rays on enhanced proliferation of irradiated cells [25]. The observed effects of low concentrations of these extracts on one of the main components of the immune response point to the possibility of their use to enhance immunity. It would be interesting to investigate their action towards different PBMC subpopulations and elucidate the potential mechanisms through which they stimulate proliferation. The possible immunostimulatory effects of extracts at lower concentrations might be explained by a modulation in lymphocyte cytokine production, including IL-2, IFN-γ, as well as IL-4 and IL-6. The immunoregulatory actions of phenolic compounds, such as quercetin, kaempferol and apigenin, have been reported [26-28]. Considering the presented results, the effects of the extracts on PBMC might be mediated through NF-κB.

Examination of *in vitro* cytotoxicity revealed that extracts 1 and 2 might be a significant source of novel promising anticancer compounds in view of their pronounced cytotoxic activities against HeLa, Fem-x and especially against K562 cells, as well as their high selectivity in the antitumor actions against cancer cells in comparison to healthy PBMC. Chemical analyses of the *Helichrysum zivojinii* extracts showed the presence of phenolic compounds whose antitumor potential has already been documented. The bioactive flavone apigenin that was found in extracts 2–5 has been reported to exhibit anticancer activities against different types of malignant cells including breast, cervical, ovarian, prostate, colon, gastric, liver and lung cancers, as well as skin and thyroid cancer, diverse hematological malignancies and neuroblastoma [28] and references cited therein]. The cytotoxic activities of extracts 2–5 may be at least in part due to the flavonoid naringenin. This flavonoid has been shown to exert cytotoxicity towards various malignant cell lines, such as breast cancer cell lines (MCF-7, MDA-MB-231), cervix adenocarcinoma (HeLa), liver cancer (HepG2, Hep3B, Huh7), pancreas cancer (PK-1), colon cancer (Caco-2), stomach cancer (KATOIII, MKN-7) and leukemia cells (Jurkat, HL-60, U937, NALM-6, THP-1) [29-31]. Additionally, the cancer-preventive and cancer-suppressive properties of quercetin, whose *O*-glycosides were identified in extracts 3, 4 and 5, have been documented as well [32]. The antiproliferative and pro-apoptotic effects of quercetin were shown against the HeLa cell line [33].

Morphological analysis of the mode of HeLa cell death, together with the cell cycle analysis, showed that the treatment of HeLa cells with higher concentrations of the examined extracts induced apoptotic cell death. To confirm the pro-apoptotic action of the tested *Helichrysum zivojinii* extracts and to identify the caspases implicated in the employed apoptotic pathways, specific caspase inhibitors were used (Z-DEVD-FMK, Z-IETD-FMK, Z-LEHD-FMK). A prominent decrease in the percentages of subG1 apoptotic HeLa cells after treatments with each of the tested extracts in combination with specific caspase inhibitors compared to the percentages of subG1 cells after treatments with only the corresponding extracts, indicates that each of the five extracts induced apoptosis through the activation of caspase-3, the main effector caspase, as well as through the activation of caspase-8 and caspase-9. We conclude that the constituents of the *Helichrysum zivojinii* extracts triggered apoptosis in HeLa cells through the intrinsic pathway mediated by caspase-9, and the extrinsic pathway mediated by caspase-8. In addition, the crosstalk between these two apoptotic pathways should also be considered. Due to their ability to promote apoptotic cell death in cancer cells, the investigated extracts and their constituents may have significant anticancer potential. It is worth noting that antitumor drugs that induce apoptosis and thereby suppress the further growth of tumors play important roles in the clinical treatment of malignancies. In addition to the pronounced inhibition of proliferation and survival of target malignant cells, the lower cytotoxicity of *Helichrysum zivojinii* extracts against healthy PBMC is a promising lead for future studies.

## Conclusions

Data from this *in vitro* study clearly demonstrate the prominent antitumor potential of five extracts prepared from the endemic plant species *Helichrysum zivojinii,* which can be attributed to their selective and pronounced antiproliferative and pro-apoptotic actions towards specific malignant cells in comparison to healthy PBMC. Prospective cancer-suppressive effects of the tested extracts should be further evaluated in *in vivo* experiments.

## Competing interests

The authors declare that they have no competing interests.

## Authors’ contributions

IM performed all analyses of the anticancer properties of investigated extracts, interpreted obtained data and wrote the first and last version of the manuscript. IA participated in design of the study, prepared extracts, performed chemical characterization, interpreted data and wrote the part of the manuscript. ŽŽ participated in acquisition and analysis of data. MJ carried out chemical analyses of the extracts. VV and SM participated in design of the study and interpreted obtained data. ZJ designed the research on anticancer properties of tested extracts, interpreted obtained data, participated in writing the manuscript and critically revised the manuscript. All authors have read and approved the final version of the manuscript.

## Pre-publication history

The pre-publication history for this paper can be accessed here:

http://www.biomedcentral.com/1472-6882/13/36/prepub
